# Surgery of the Spine and Cord

**Published:** 1895-08-31

**Authors:** 


### PROGRESS IN SURGERY.

### SURGERY OF THE SPINE AND CORD.

(*Continued from page 362.*)

**Tumours of the Cord.—Diagnosis.**—As illustrating the difficulties in the diagnosis of spinal cord lesions, Pfeiffer[30] reports a case which presented symptoms indicative of a tumour of the cord about the level of the sixth and seventh dorsal vertebræ—pain, tenderness, transient bladder disturbances, exaggerated reflexes in the lower limbs, trailing of the left foot and retarded sensation. On exploration no tumour was found, and the patient died two months later of cystitis and bed sores. On post mortem examination myelitis in the mid-dorsal region, with ascending and descending degeneration were found. Another case communicated by Gieson and Fisher,[31] in which the history and general symptoms strongly suggested locomotor ataxia, proved to be one of extra-medullary gliosarcoma situated centrally in the region of the second lumbar vertebra and extending three inches down into the cauda equina. In spite of its involving several nerve cords there was no pain. In a case quoted by Bennet,[32] the prominent symptoms of a spinal tumour were neuralgic pain in the leg, greatly increased on movement of the body, with patchy anæsthesia. A recent attack of influenza led to errors in diagnosis, the pain being looked upon as one of the nervous sequelæ of that disease. Bennet considers pain on movement a valuable point in diagnosis as between malignant disease and caries, in the former of which it is well marked, in the latter slightly. He also calls attention to the frequency with which malignant disease of the spinal column appears secondarily to scirrhus of the breast.

Cases of *coccygeal dermoid cyst* are reported by Wyeth, Abbe, Meyer, and Briddon.[33] Points of interest in connection with them are (1) their comparative frequency; (2) their tendency to suppurate; (3) the difficulty of getting healing by first intention after excising them (doubtless on account of the close proximity of the anus); and (4) the tendency they have to undergo epitheliomatous degeneration.

Sanger and Krause[34] report a case in which a spindle-celled sarcoma was removed from the spinal cord. The patient, 42 years of age, fell on his left side, and for nine months thereafter complained of lancinating pains, first on the left, and later on both sides of his chest. Subsequently he had girdle pains, paraplegia, contractures of legs and analgesia. There were no bed sores, but the skin and tendon reflexes of the lower extremity, as well as the motor power of the bladder, were lost. There was tenderness on pressure over the spines of the seventh to tenth dorsal vertebræ. Anti-syphilitic treatment was unavailing, and an operation revealed the tumour in the region of the sixth dorsal vertebræ, between the dura and the bone. It was removed without difficulty, and with but slight hæmorrhage. Death followed on the fourth day from bronchitis and hypostatic pneumonia.

**Spina Bifida.**—*Apropos* of a successful case of excision of a very large spina bifida, H. O. Marcy[35] contributes a valuable paper on the surgical treatment of this condition. His patient, a young lady of eighteen, had been born with a soft swelling in the lumbar region about the size of half an orange, which had steadily grown until it had reached the formidable proportions of nineteen inches by eighteen inches, with a circumference of twenty-nine inches, and was dangerously near rupturing. On operating, about a gallon of clear, colourless fluid was withdrawn. The cauda equina was spread out on the wall of the sac. In discussing the treatment of this condition, Marcy suggests the analogy of the radical cure of hernia—the isolation and removal of the sac (having first ascertained its contents), and the reinforcement of the structures in a way to prevent as far as possible the return of the tumour. Failures are always due to sepsis. The higher the opening in the column the better is the prognosis as to return, because the hydrostatic pressure is less. The direct connection between the fluid in the sac and that surrounding the great nerve centres has to be borne in mind, in emptying the sac, to avoid too rapid withdrawal of the fluid and thus interfering with the cerebral vascular arrangements. To prevent this the fluid should be withdrawn through a small cannula, the head being lowered meanwhile. Its bearing on the spread of sepsis is obvious. The operation is performed by exposing the sac by elliptical incisions, after which it is emptied, and any nerve elements found in it restored to their place inside the spinal canal. The base of the sac is then completely closed by absorbable stitches, cut off, and anchored to the fascia of the quadratus muscles. No attempt is made to obtain a bony covering by transplanting periosteum. The wound is sealed with iodoform collodion and wool.

Van Hook[36] also advocates treatment by excision as against the injection method, which he considers "a relic of a past surgical age." He quotes statistics which show a percentage of 76·9 recoveries by the open method, and 65 by injection.

Successful excisions are recorded by Young[37], and Ramsay,[38] whose patient nine months after operation shows no tendency to recurrence and no bad symptoms.

Against these satisfactory cases must be put the statement of Broca,[39] who reports ten cases of excision. Three died from septic infection of the operation wound. Only one of the seven who survived was known to be quite well at the end of twelve months; three had not been seen; and the other three suffered from hydrocephalus, which, in the author's experience, is often associated with spina bifida, and is the commonest cause of failure after the operation. He advocates operation only when the tumour has burst or is on the point of bursting. Tédenat[40] also takes a less hopeful view of the operation than the American surgeons quoted. One of his cases died immediately on the puncture of the sac with a Pravaz syringe. By Morton's method one of two patients was cured, the other suffered from convulsions and hydrocephalus two months after. After excision one patient developed hydrocephalus, one remained completely paralysed, and a third recovered.

Durham[41] reports a case in which Morton's method failed to bring about a complete cure, and in which subsequent repeated tappings without injection have been equally futile. Woodyard's[42] case was cured by the use of the iodine fluid.

[30] Neurol. Centralbl., Nov., 1894. [31] Med. Week, Dec., 1894. [32] Clin. Jour., Oct., 1894. [33] Ann. Surg., Aug., 1894. [34] Münch. Med. Woch., Nov., 1894. [35] Ann. Surg., Mar., 1895. [36] North Amer. Practitioner, Vol. II., No. 5. [37] Therapeutic Gazette, Ap., 1895. [38] M. B. J., June, 1895. [39] Sem. Med., Oct., 1894. [40] Amer. Jour. Med. Sc., June, 1895. [41] Clin. Jour., May, 1895. [42] Virginia Med. Monthly, May, 1894.

